# Supplementary material for: Cross-family and phage-specific gene requirements for Klebsiella infection revealed by scalable RB-TnSeq genetic screens
Source: PLoS Genet. 2026 Jul 20;22(7):e1012233. doi: 10.1371/journal.pgen.1012233 (PMC13412088; doi:10.1371/journal.pgen.1012233)
Supplement: Attachment — Submitted filename: Response to reviewers.docx [file pgen.1012233.s020.docx]

**Reviewers' comments are in italics and author responses are in light-blue**

*Reviewer #1: In “Cross-family and phage-specific gene requirements for Klebsiella infection revealed by scalable RB-TnSeq genetic screens”, Gittrich et al. perform a series of barcoded Tn-seq screens to identify fitness determinants for a panel of 25 dsDNA Klebsiella phages that infect the acapsular environmental Klebsiella species M15aI. The stated goal of the study was to identify genes required for infection of each phage and from there to discern broader patterns comparing across the diverse phages. This type of screen is successful at identifying phage receptors or genes that encode regulators of phage receptors, as has been seen in previous similar efforts. Most other bacterial genes required for phage replication are likely essential, e.g. any of the central dogma processes that phages hijack and commandeer to replicate themselves. A screen like this will also not identify any genes involved in bacterial antiphage defenses – which also contribute to host range – as the phages used here are by definition are not susceptible to any defenses present in this particular strain.*

Response: We thank the reviewer for the comments. Here we present a revised version of our prior submission with additional experiments, complete re-organization and presentation of the work. We also provide a detailed response to each of the reviewer comments. We thank the reviewer for the opportunity to clarify two points that we believe merit further discussion.

**On the characterization of this study relative to prior work:** The reviewer frames this study as one of several “similar” efforts without acknowledging its scale or the novelty of the biological system. To our knowledge, this is the largest RB-TnSeq receptor mapping study conducted in any single bacterial host outside of *E. coli,* encompassing 25 phages spanning five families across 213 parallel assays. The only comparable study in scale is Moriniere et al. (2026, https://www.biorxiv.org/content/10.64898/2026.04.02.716166v2), which screened 255 *E. coli* phages using the same approach and was published while this manuscript was under review. In *Klebsiella* specifically, no prior genome-wide screen had been conducted across more than two phages simultaneously (as we detail below and also in the main text), and no study had connected a diverse phage collection to receptor gene dependencies comparatively across phage families and genera. The scale of this study enabled us to study the taxonomy-level pattern analysis that no prior work has been performed.

**On the claim that RB-TnSeq cannot identify antiphage defense systems and therefore provides an incomplete view of host range determinants:** We thank the reviewer for raising the point about antiphage defense systems, as it allows us to clarify an important aspect of the biology underlying RB-TnSeq datasets that we believe is worth addressing explicitly in the context of this study.

The reviewer's argument rests on the assumption that antiphage defense systems present in this strain would prevent infection by the phages used in this study, and that their absence from the RB-TnSeq hits therefore represents a blind spot of the method. We believe this reasoning conflates two distinct questions. The first is whether defense systems can in principle be detected by RB-TnSeq. They can, provided that the phage being screened is sensitive to the defense system in question, in which case disruption of the defense gene would confer a measurable fitness advantage on the mutant. The second and more fundamental question is why defense systems do not dominate RB-TnSeq datasets in practice, and the answer has less to do with the limitations of the method than with the biology of defense systems at native expression levels.

As the phage research community knows, the overwhelming majority of published studies characterizing antiphage defense systems have done so using multicopy plasmid-based overexpression systems rather than in their natural single-copy genomic context. When assessed at native chromosomal copy number and expression levels, the protective effect of most individual defense systems is substantially attenuated relative to overexpression conditions (unpublished data). To our knowledge, only a single published study has assessed the contribution of natively encoded, chromosomally integrated defense systems to phage resistance at the population level, and that study demonstrated that the fitness advantage conferred by most individual defense systems in their natural context is (<https://www.biorxiv.org/content/10.64898/2025.12.14.694197v1>) modest and highly condition-dependent. This is entirely consistent with the observation that defense system genes do not emerge as dominant hits in RB-TnSeq datasets across multiple published studies in different bacterial species (manuscript in preparation, Mutalik Lab). Receptor loss confers complete or near-complete resistance by abolishing phage adsorption at the earliest step of infection, producing large and reproducible fitness advantages that dominate pooled fitness assays. Defense system disruption, by contrast, typically produces only a partial reduction in infection efficiency at native expression levels, and effect sizes may fall below the detection threshold of a pooled assay. Therefore, we think that the absence of defense system hits across this and comparable published datasets is most parsimoniously explained by the small effect sizes of natively encoded defense systems rather than by a fundamental limitation of the RB-TnSeq approach, and that this interpretation is consistent with the available literature on defense system activity in genomic context.

*Reviewer #1: Thus, as expected, most of the 42 hits identified here are in genes that are already associated with phage infection, as these are directly or indirectly involved in producing or regulating production of phage receptors. The authors do not follow up at all on any of the unexpected genes that they hit. From a technical standpoint, the Tn-seq has been carefully carried out with sufficient replicates, and clearly represents a laborious effort. However, despite the scale of the screen, it does not appear we have learned much new biology. One lens through which this work could be viewed is as a high throughput method for mapping phage receptors; however, the authors do not take this angle. I also do not think that based on this study we can conclude very much about infection strategies or requirements (outside of receptors), as many of the genes required for infection – as I noted above – are likely essential. Yet as I elaborate on below, of the potentially unexpected genes identified, none were followed up on in much depth. There are a few interesting hits that are not obviously receptors: NlpD, TolR. The authors discuss possible well thought through and referenced hypotheses for each at length, but not only do they not test any of these, they do not even validate these hits via knock out or complementation. Similarly, regarding the regulators they hit, they speculate that e.g. SdiA might alter phage adsorption via increases in biofilm formation when it is deleted; however, this is never actually tested.*

Response: We appreciate the acknowledgement of the technical rigor of the RB-TnSeq approach and the enormous time these global screens, when done at scale and design as presented here, take. In fact, a single PhD student as lead author drove most of this work learning and establishing the method from scratch in a non-genetics lab to develop this body of work, so we simply ‘timed out’ on the extensive follow-up experiments the reviewer is suggesting. While we felt that advancing this large-scale phage-host system from virtually zero knowledge to that laid out here was already an impressive advance in our understanding and an incredible foundation for researchers to build from, we also take the substantive criticisms the reviewer highlights seriously. It’s not that we did not see value to such experiments, rather we felt the scope of work was already immense here and generated a formidable list of excellent hypotheses for the field to test. Seeing these reviews, however, we have brought in a team of new students to try to help deliver on the scale of additional work requested.

We address each point below and hope to demonstrate that the reviewer’s criticisms underestimate both the novelty of the findings and the significance of the experimental system.

(1) The reviewer's assessment that little new biology has been learned rests on the premise that most of the 42 hits were already known to be associated with phage infection. We respectfully contest this characterization. Prior knowledge of putative phage receptors in *Klebsiella* was extraordinarily limited before this study (Table S7). The most comprehensive prior receptor study in this genus, Dunstan et al. (2023, [PMID: 37224021](https://www.zotero.org/google-docs/?y91SUl)), characterized two receptors for two phages in a single capsulated strain and themselves noted in their introduction that known *Klebsiella* phage receptors in the literature were limited to OmpK36 for three phages, FepA for one phage, and LPS core or O-antigen for a small number of others. That represents the entire state of receptor knowledge in this genus accumulated over decades of work, with no study having assembled a diverse phage collection to map receptor gene dependencies systematically and comparatively across them. The present study does precisely this for 25 phages spanning five families, providing the first systematic comparative map of host factors required for phage infection across a diverse *Klebsiella* phage collection.
(2) We wish to emphasize a point that is central to interpreting RB-TnSeq fitness scores as evidence in their own right, independent of follow-on clonal validation. In our saturated transposon library, each non-essential gene is disrupted by an average of six and up to approximately twenty independent transposon insertions, each carrying a unique barcode tracked separately throughout the experiment. The gene-level fitness score reported by RB-TnSeq therefore represents an internal consensus across multiple independent loss-of-function clones within the same experiment, and a gene passing our stringent thresholds reflects reproducible enrichment across many independently created disruption mutants. This level of within-experiment replication is rarely achievable through traditional single-knockout approaches and constitutes a form of “built-in validation” intrinsic to the method. Across our prior published RB-TnSeq studies, more than 200 genes have been validated by individual knockout experiments without a single case of a high-confidence RB-TnSeq hit failing clonal validation, establishing a strong empirical track record for the reliability of this approach (PMID 25968644, 38870284, 40068681, 41984924, 41477825, 41025825, 40981468, and more, including Moriniere et al. 2026).
(3) Nonetheless, we recognize the value of gene-specific validation experiments in this system, and in the revised manuscript we have substantially expanded the experimental validation section. Three new co-authors have joined the study to contribute these experiments, and Dr. Mutalik has been elevated to co-corresponding author reflecting his central intellectual and experimental contribution to this revised work. Confirmation of gene requirements for phage infection was carried out by efficiency of plating comparing wild-type to individual sequence-confirmed knockout and complemented strains. In addition to the LPS outer core biosynthesis genes *wabI*, *wabN*, and *wabH* validated in the original submission, the revised manuscript includes new validation experiments for OmpK36 as a primary cross-family receptor in an acapsular host, FadL and PqqU as functional receptors in *Klebsiella* for the first time, and PhoQ, RS16715, and RS24775 as novel host factors influencing phage infection, none of which had any prior association with phage infection in any bacterial system. We hope these extensive additional validations, combined with the internal replication inherent to the RB-TnSeq approach, demonstrate the rigor and biological novelty of the findings reported in this study.
(4) Having discussed the reviewer's framing suggestion amongst our team, we agree that positioning this work explicitly as a high-throughput receptor mapping study strengthens the presentation and we have now revised the manuscript accordingly throughout the Introduction, Results and Conclusion. The receptor map produced here spans LPS outer core biosynthesis, outer membrane porins, fatty acid transport, cofactor biosynthesis, and two-component signaling across 25 phages and five families, representing the first systematic, cross-family receptor map in Klebsiella and providing a community resource for linking phage genomic diversity to functional host interaction space.
(5) In this revised manuscript, we report Individual sequence-confirmed knockout strains with complementation were generated and validated for a total of eight genes. These comprise

- the LPS outer core biosynthesis genes *wabI*, *wabN*, and *wabH*, each validated by individual knockout, complementation, and adsorption assays;
- the outer membrane porin *ompK36*, validated by individual knockout, complementation, and efficiency of plating;
- the outer membrane fatty acid transporter *fadL*, validated by individual knockout, complementation and efficiency of plating;
- the pyrroloquinoline quinone biosynthesis gene *pqqU*, validated by individual knockout and efficiency of plating;
- the predicted inner membrane protein RS24775, validated by individual knockout, complementation and efficiency of plating;
- *dsbA*, validated by individual knockout, efficiency of plating, and liquid growth inhibition assays.

(6) We also wish to address the implicit suggestion that studying phage infection in an acapsular strain is a limitation relative to capsulated clinical strains. The acapsular host choice was made on deliberate scientific grounds and the findings it produces are directly relevant to capsulated clinical populations, not only to environmental acapsular strains. As we mention this in the revised introduction section, capsule loss is the single most common mechanism by which *K. pneumoniae* evolves resistance to phage predation, arising in more than 97% of phage-resistant mutants in controlled experiments. When capsulated clinical strains shed their capsule under phage selection pressure, the receptor landscape they present to phages becomes precisely the one characterized in this study. The LPS outer core biosynthesis genes *wabI*, *wabN*, and *wabH*, *ompK36*, *fadL*, and *pqqU* identified and validated here therefore represent not only the receptor biology of environmental acapsular populations but the secondary receptor layer that becomes exposed and therapeutically important in capsulated clinical strains once capsule loss occurs. Every prior transposon screen in *Klebsiella* was conducted in capsulated strains, where the capsule signal dominates and these secondary/terminal receptor structures are invisible. The present study provides the first systematic characterization of the post-capsule-loss receptor landscape in Klebsiella, information that is directly actionable for the design of phage cocktails intended to cover the resistance escape variants that inevitably arise under therapeutic phage pressure.
(7) Regarding the non-receptor hits, the reviewer raises the concern that genes required for phage replication are likely essential and therefore undetectable by this approach. This is correct as a general principle and is explicitly acknowledged in the revised manuscript. The 17 non-receptor genes identified here are by definition non-essential for growth under the assay conditions, as they were recovered as viable transposon mutants competing in the pooled library. The reviewer's concern therefore applies to genes absent from the dataset rather than to those present within it. For the hits detected, each is supported by reproducible fitness signals across multiple independent transposon insertions, with a typical range of 3 to 20 independent mutants per gene contributing to each fitness score. We have revised the manuscript to present these consistently as high-confidence candidates for future mechanistic investigation, which is the appropriate and standard treatment for novel screen-level findings in this field. This is evidenced by comparable published RB-TnSeq phage-host interaction studies, specifically Mutalik et al. (2020, *PLOS Biology*) and Adler et al. (2021, *Microbiology*), both of which used the same approach to identify bacterial host factors required for phage infection in *E. coli* and *Salmonella* respectively, reported novel hits without exhaustive single-gene mechanistic follow-up, and are widely cited community resources in the phage-host interaction field. Rotman et al. (2024) applied the same transposon screen approach to *K. pneumoniae* without individual knockout mechanistic dissection of receptor genes and was accepted as a significant contribution in *Cell Host and Microbe*. These precedents establish that screen-level identification of phage infection determinants, with selective experimental validation of the most unexpected and informative hits, represents the accepted standard for this class of study.
(8) Also, we note that the term ‘essential’ overstates the evidential standard supported by RB-TnSeq fitness scores, particularly for genes such as *dsbA* where disruption yields partial rather than complete resistance (compared to receptor mutations), and for genes where clonal knockout validation has not been performed. We have revised the manuscript throughout to replace the word essential with more appropriate language that accurately reflects the nature of the evidence. Genes with validated complete resistance phenotypes are described as functional receptor determinants or confirmed host factors required for infection. Genes with validated partial resistance phenotypes, such as *dsbA*, are described as host factors that contribute to infection efficiency. Genes identified by screen signal alone without clonal validation are described as high-confidence candidates associated with phage infection, supported by reproducible fitness signals across multiple independent transposon insertions. This three-level distinction is now applied consistently across the abstract, Introduction, Results, and Conclusion sections. We believe this revised language accurately represents the graduated nature of the evidence and avoids the overstatement that the term essential implies.
(9) Finally, our analysis reveals that phage genus rather than family is the stronger predictor of host gene dependency profiles, a pattern-level result established by PERMANOVA across 25 phages spanning five families that no single phage-host study can produce. This finding has direct practical implications for the functional annotation of the rapidly expanding repositories of uncharacterized Klebsiella phages and for the rational design of phage cocktails where covering distinct receptor dependencies is a key criterion for minimizing cross-resistance. We have strengthened this section in the revised manuscript to ensure its novelty and practical significance are presented with appropriate clarity.

*Reviewer #1:One hit that was not an obvious receptor is DsbA, but notably, DsbA was identified in an earlier, similar Tn-seq study performed in Acinetobacter where the authors delved into how it affects capsule synthesis; here, we have no further information about what might be happening. It seems potentially interesting that DsbA was hit in a study with a capsulated bacterium as well as here; do they signal through a similar regulator that alters the cell surface here as well?*

Response: We thank the reviewer for this insightful observation. The parallel between the DsbA hit in *Acinetobacter* and the present study is indeed noteworthy and your comments have spurred us to more deeply evaluate it for consideration in the revised manuscript.

In the *Acinetobacter* study, *dsbA* disruption was associated with truncation of the capsular polysaccharide, which paradoxically increased phage adsorption by exposing underlying surface receptors, resulting in a gain-of-infectivity phenotype. The mechanism therefore operated through capsule-dependent surface remodeling. In *Klebsiella* sp. M5al, which is naturally acapsular, *dsbA* loss produces a partial resistance phenotype rather than increased susceptibility, indicating that a capsule-dependent mechanism cannot account for the effect observed here. The two observations are therefore phenotypically opposite in direction, which argues against a shared regulatory pathway acting through capsule modification.

Instead, we favor the mechanistic interpretation in our system that DsbA influences phage susceptibility through its well-established role in the oxidative folding of outer membrane proteins and periplasmic components. DsbA catalyzes disulfide bond formation in periplasmic and outer membrane proteins, and its disruption is known to impair the folding and surface presentation of multiple outer membrane proteins in gram-negative bacteria. In *Klebsiella* sp. M5al, where LPS outer core and outer membrane porins serve as the primary receptor structures, DsbA-dependent folding of one or more of these surface components could plausibly influence their accessibility or structural integrity as phage receptor targets. The co-occurrence of *dsbA* and *cydA/cydB,* encoding cytochrome bd oxidase, and the fitness signals across the same phages noted in the manuscript are consistent with this interpretation, as cytochrome bd oxidase contributes to the reducing power that supports DsbA activity.

We acknowledge that whether a common regulatory pathway involving surface remodeling underlies the DsbA effect in both *Acinetobacter* and *Klebsiella* systems remains an open question. We have added a note in the revised manuscript flagging this as a direction worth pursuing in future work, and we thank the reviewer for drawing attention to this potentially informative comparison.

*Reviewer #1: Finally, they identify 3 genes never before associated with phage infection – a stated objective of their study – but do not follow up on these at all.*

Response: We thank the reviewer for this comment and agree that the three genes with no prior association with phage infection in any bacterial system, namely *mtlD*, RS22875, and *tatC*, represent new findings that warrant discussion. We have expanded the treatment of these genes in the revised manuscript, presenting each with a brief description grounded in published biology and framing them explicitly as high-confidence candidates supported by reproducible fitness signals across multiple independent transposon insertions. We wish to offer a broader reflection on the challenge the reviewer raises. Functional follow-up on novel screen hits is highly productive when the hit encodes a surface-exposed receptor, because the phenotype of a receptor knockout is large, binary, and mechanistically interpretable through a small number of well-established assays. For hits encoding intracellular functions of unknown connection to phage biology, the path from screen signal to mechanistic understanding is considerably less predictable. The history of genetics broadly is replete with cases where disruption of an intracellular gene produces a reproducible phenotype whose mechanism resists straightforward dissection, and phage-host interaction genetics is no exception. The modest and phage-specific effect sizes associated with these genes suggest indirect or context-dependent contributions to infection that are unlikely to yield to simple knockout-complementation experiments alone and would require dedicated mechanistic investigation beyond the scope of this study.

This reflection informed our decision, taken in response to the reviewer's broader suggestion, to refocus the manuscript explicitly on its strongest contribution, namely the high-throughput systematic mapping of phage receptors across a diverse *Klebsiella* phage collection. Receptor identification is precisely where this approach excels and where follow-up validation is most tractable, as demonstrated by the eight validated genes reported in the revised manuscript. The three novel intracellular hits are presented as the first reported associations of these genes with phage infection in any bacterial system and as tractable targets for future dedicated investigation, while the primary narrative of the revised manuscript centers on the receptor map and the taxonomy-level patterns that emerge from it.

*Reviewer #1: None of hits, except for DsbA and WabI, were validated in single gene deletion assays or through complementation.*

Response: In the revised manuscript, we have substantially expanded and restructured the experimental validation section to present the full scope of the validation experiments with greater clarity. As mentioned above, in this revised manuscript, we report Individual sequence-confirmed knockout strains with complementation were generated and validated for a total of eight genes. These comprise ***wabI*, *wabN,* and *wabH*; *ompK36*; *fadL*; *pqqU*; *RS24775*; and *dsbA*.** We hope the revised presentation makes the breadth of the experimental follow-up clear and addresses the reviewer's concern.

*Reviewer #1: They perform some analyses looking for patterns among and between phages, and find that consistent with many previous studies, phage receptor usage mediated by phage tail fiber variation is responsible for most of the variation observed. Thus despite their contention that their work with acapsular strains can identify a broader set of host range determinants, which may or may not be the case, they did not really leverage this stated advantage to learn anything more about these determinants.*

Response: We thank the reviewer for this comment and respectfully disagree with the characterization that the pattern-level findings are merely consistent with previous studies and add little beyond what was already known.

Prior studies establishing that tail fiber variation drives host range differences have largely done so through single-phage comparisons or small panels, typically examining two to four phages in pairwise or small-group comparisons within a single genus or family. A notable recent exception is Moriniere et al. (https://www.biorxiv.org/content/10.64898/2026.04.02.716166v1.full), who reported genome-wide screens across 255 *E. coli* phages and demonstrated that receptor identity can be predicted from genome sequence using machine learning. In the present study we ask whether phage taxonomic rank, at both family and genus resolution, predicts the full profile of host gene dependencies and demonstrates for the first time in *Klebsiella* that genus-level classification carries quantifiable predictive power for genome-wide infection strategy. The PERMANOVA analyses show that genus explains significantly more variance in host dependency profiles than family across five independently tested families, a result that emerges specifically from the comparative architecture of screening 25 phages in parallel and that no smaller-scale study could produce.

Regarding the reviewer's suggestion that the acapsular host advantage was not fully leveraged, we have now performed extensive additional experimental validation for high-scoring genes in our genetic screens. We have listed these validations in the above comment. Characterizing these host factors, therefore, required the deliberate choice of an acapsular host, and the validated findings are a direct consequence of that choice. We have strengthened this argument in the revised manuscript throughout (and in the early part of our response in this document) to ensure the connection between the experimental design and the specific findings is explicit and clearly communicated throughout.

*Reviewer #1: Minor point: the figures were extremely pixelated to the point that it was hard to interpret the data in some cases (e.g. the PCA analysis I could barely tell apart the different shapes of the same color).*

Response: We are sorry to hear that the originally submitted manuscript suffered from pixelated figures. We have now evaluated all figures to ensure they are of high quality in this revised manuscript.

**—---------------------- Reviewer #2 —-------------------------**

*Reviewer #2: Using a genome-wide RB-TnSeq approach in Klebsiella sp. M5al, this study identifies 42 bacterial genes required for phage infection across diverse phage families. The work demonstrates that bacterial gene requirements cluster significantly by phage taxonomy, while revealing that even closely related phages can exhibit distinct infection strategies due to tail fiber variation and differential reliance on intracellular host factors. The manuscript is original, well written, and supported by extensive raw and processed data.*

Response: We thank Reviewer 2 for the thorough and generous assessment of this manuscript and for the recognition of the originality and rigor of the work. We are pleased that the core findings, including the taxonomy-dependent clustering of host gene dependencies and the intra-genus divergence in infection strategy, were clearly communicated. In the revised manuscript we have further strengthened the presentation of these findings in response to comments from the other reviewers including additional experimental validation of high scoring host factors and we hope the revised version addresses all remaining concerns to the reviewer's satisfaction.

*Specific comments

Reviewer #2: Line 76: The term phage tail fibers is too restrictive, as not all receptor-binding proteins adopt a fibrous morphology (e.g., tail spikes). Phage receptor-binding proteins would be a more accurate general term.*

Response: Replaced “tail fiber” with “phage receptor binding proteins” at (prior) line76 and in other instances, specifically when referring to the tail fiber region involved in receptor binding.

*Reviewer #2: Line 96: Please elaborate on the RB-TnSeq principle for readers unfamiliar with the method. Note that if a phage depends on a bacterial protein essential for host survival, this dependency cannot be detected with RB-TnSeq. This limitation seems currently not acknowledged.*

Response: We thank the reviewer for this helpful suggestion. We have added text to the Introduction elaborating on the RB-TnSeq principle for readers unfamiliar with the method, including a description of how gene-level fitness scores are calculated from barcode abundance changes across multiple independent transposon insertions. We have also explicitly acknowledged the limitation noted by the reviewer, namely that RB-TnSeq relies on the survival and recovery of transposon mutants and therefore cannot detect dependencies on genes that are essential for bacterial growth under the assay conditions. Genes essential under all conditions are absent from the mutant library and cannot be assessed by this approach. This limitation is now stated clearly in both the Introduction and the Results section, where we note that the 42 genes identified here represent the non-essential component of the host genetic landscape governing phage infection and that essential host factors co-opted during phage replication, such as components of the core transcription and translation machinery, fall outside the detectable space of this assay. We believe this qualification appropriately contextualizes the scope of the findings without diminishing the value of the receptor and host factor dependencies that the screen successfully identified.

*Reviewer #2: Additionally, the dataset reflects infections in rich medium; nutrient composition likely influences host gene dependencies. In nutrient-poor conditions, dependencies may increase, but more bacterial genes will become essential and can’t be discovered with this approach.*

Response: We thank the reviewer for raising this important point. We agree that the nutrient composition of the assay medium is a relevant contextual variable that shapes the gene dependency landscape in two complementary ways. First, genes that become essential only under nutrient-limiting conditions will not be represented in the mutant library under rich medium conditions and are therefore outside the detectable space of this assay. Second, genes involved in nutrient acquisition or metabolic adaptation that are dispensable in rich medium may become important phage infection determinants under nutrient-poor conditions, where their disruption could alter host physiology in ways that influence receptor expression, surface composition, or phage replication efficiency. Both of these effects mean that the 42 genes identified here represent the host gene dependency landscape specifically under rich medium conditions, and that screens conducted under different nutritional regimes would likely reveal a partially distinct and potentially expanded set of dependencies. We have added a sentence to the revised manuscript explicitly acknowledging that the screen was conducted in rich liquid medium and that host gene dependencies identified under these conditions may not fully reflect those operating in nutrient-limited or environmentally relevant contexts, including the soil environments where *Klebsiella* sp. M5al and its phages naturally co-exist. We thank the reviewer for prompting this clarification, which we believe appropriately covers the scope and transferability of the findings.

*Reviewer #2: Was a phage-free control included over the same time interval? Some enriched mutants, particularly those difficult to interpret, might reflect general growth advantages under the experimental conditions (LB, shaking, 30°C). These methodological limitations should be explicitly discussed.*

Response: A phage free control was run with everything RB-TnSeq assay and the averages of the phage free control are shown in Figure 2. We added a sentence in the Figure legend for clarity, “We also included ‘no-phage’ culture where SM buffer was mixed with the library.”

*Reviewer #2: Line 135: Justify the selection of the 24 + 1 phages. The choice appears pragmatic based on availability (which is understandable) rather than diversity-driven (e.g., absence of Casjensviridae or Peduoviridae; see Fig. 1).*

Response: We thank the reviewer for this observation. The reviewer is correct that the phage collection reflects the isolates capable of infecting *Klebsiella* sp. M5al rather than a diversity-driven selection designed to represent all known *Klebsiella* phage families. We have revised the manuscript to state this explicitly, noting that the 25 phages used in this study represent all double-stranded DNA isolates capable of productively infecting this host that were available at the time of the screen, and that the collection was therefore host-range defined rather than taxonomy-defined. The absence of families such as *Casjensviridae* and *Peduoviridae* reflects the inability of available isolates from those groups to infect *Klebsiella* sp. M5al under the conditions tested rather than a deliberate exclusion. We note that this is an inherent feature of any screen conducted in a single host strain, and that the five families represented in this collection nonetheless capture substantial genomic and morphological diversity, including three of the most prevalent families among *Klebsiella* phages and two recently described novel families. Expanding the host range of future screens to include additional *Klebsiella* strains susceptible to a broader range of phage families would be a valuable direction for extending the receptor map produced here to underrepresented phage groups. We thank the reviewer for prompting this clarification, which we have incorporated into both the Methods and the Discussion sections of the revised manuscript.

*Reviewer #2: Discussion on Autographiviridae: These phages are often described as relatively host-independent due to their RNA polymerase, which explains their frequent use in engineering projects for host swapping. This trend is partially reflected in the dataset, with the three Autographiviridae showing reduced dependence on intracellular proteins compared to Drexlerviridae and some Straboviridae. However, RS11475 is an exception for Autographiviridae and is not discussed.*

Response: We appreciate this insightful comment. Members of the *Autographiviridae* encode their own virion-packaged RNA polymerase, which allows them to initiate transcription of their genome independently of the host transcriptional machinery immediately upon infection. This self-sufficiency in early transcription is widely considered to reduce their dependence on host intracellular proteins relative to phage families that rely on host RNA polymerase for early gene expression, and this pattern is reflected in our dataset, where the three *Autographiviridae* phages show reduced fitness hits for intracellular host factors compared to *Drexlerviridae* and several *Straboviridae* phages.

Regarding RS11475, we thank the reviewer for drawing attention to this exception. RS11475 is included in the complete hit table provided in Table S7. Given the scope of this study and our decision to focus the manuscript discussion on the most broadly informative findings across the full phage panel, individual gene hits that are specific to a single phage and lack sufficient functional annotation for mechanistic interpretation have not been discussed in the main text. We acknowledge RS11475 as a candidate of interest for future investigation.

*Reviewer #2: Validation of RB-TnSeq hits: Not all targets identified and discussed are validated by knockouts (ideally complemented to exclude polar effects). The manuscript uses the term “essential” for all 42 genes (e.g. in the abstract), yet several hits confer only partial resistance and lack orthogonal validation. Essential genes imply strict necessity for infection, requiring absolute functional requirement. Figures 2 and 3 suggest context- and phage-specific fitness signals, with dsbA disruption yielding modest effects, indicating graded rather than absolute requirements. Without systematic single-gene knockouts and complementation, essentiality cannot be conclusively established.*

Response: We thank the reviewer for this precise and valid criticism. The reviewer is correct that the term "essential" overstates the evidential standard supported by RB-TnSeq fitness scores, particularly for genes such as *dsbA* where disruption yields partial rather than complete resistance, and for genes where clonal knockout validation has not been performed. We have revised the manuscript throughout to replace "essential" with more appropriate language that accurately reflects the nature of the evidence. Genes with validated complete resistance phenotypes are described as functional receptor determinants or confirmed host factors required for infection. Genes with validated partial resistance phenotypes, such as *dsbA*, are described as host factors that contribute to infection efficiency. Genes identified by screen signal alone without clonal validation are described as high-confidence candidates associated with phage infection, supported by reproducible fitness signals across multiple independent transposon insertions. This three-level distinction is now applied consistently across the abstract, Introduction, Results, and Conclusion sections. We believe this revised language accurately represents the graduated nature of the evidence and avoids the overstatement that the term "essential" implies. We thank the reviewer for drawing attention to this important point, which has substantially improved the precision of the manuscript.

*Reviewer #2: Line 410–411: The statement does not hold for KLB12 and wbiN/wbiH. Please clarify the suggestion for additional receptor usage and how this claim is derived. An additional receptor typically refers to a secondary receptor engaged after initial binding to the primary receptor. Do the authors mean two possible primary receptors instead?*

Response: We thank the reviewer for this important clarification. Disruption of *wabN/wabH* conferred only partial resistance to KLB12, indicating that while these genes support efficient infection, their loss does not fully abolish phage entry. This suggests that KLB12 can still adsorb to and infect the host without functional WabN/WabH-dependent surface structures.

We did not intend to imply a classical two-step primary/secondary receptor model, but rather to suggest that KLB12 may use multiple surface structures to support infection. In phages requiring sequential receptor engagement, loss of the primary receptor usually confers near-complete resistance. The partial resistance phenotype observed here is therefore more consistent with redundant receptor usage or alternative surface components. To avoid overinterpretation, we have revised the manuscript to state results more cautiously that the “data are consistent with potential alternative receptor usage”, without specifying a mechanistic model.

*Reviewer #2: Line 437: Clarify that “the protein itself” refers to the distal tip of the long tail fiber, not one of the three other conserved long tail fiber proteins. For reproducibility, include accession numbers for these proteins in lines 716–721. The discussion could be strengthened: the “red” KLB15 tip domain is overall more similar to the “blue” tip, suggesting horizontal transfer of the C-terminal domain, which ultimately interacts with LPS, correlating with the binding profiles.*

Response**:** We thank the reviewer for these precise and constructive suggestions, all of which we have incorporated into the revised manuscript.

First, "the protein itself" has been revised to "the distal tip of the long tail fiber" to clearly distinguish this structural element from the three other conserved long tail fiber proteins, avoiding any ambiguity about which component is being discussed.

Second, accession numbers for the distal tail fiber subunit sequences used in the phylogenetic and structural analyses have been added to the Methods section to ensure full reproducibility.

The C-terminal receptor-binding domain of KLB25 shows greater overall similarity to the blue group phages requiring *wabI*, *wabN*, *wabH*, and *wabO* than to the red group, despite KLB25 being classified within the red group based on overall tail fiber phylogeny, suggesting potential horizontal acquisition of this region consistent with its LPS binding profile. This observation is noted in the revised manuscript. The broader interpretation, that this mosaic architecture reflects how intra-genus receptor diversification can arise through domain-level horizontal transfer, is implicit in the text.

*Reviewer #2: Figure 7: AlphaFold predictions suffer from its inability to resolve relative domain positioning, resulting in curling rather than straight fibrous structures in this case. Quality metrics (pLDDT, PAE) for these predictions are missing, and the superimposition of both tail fibers is problematic. Consider alternative approaches such as RBPSeq (*[*https://www.science.org/doi/10.1126/sciadv.adv0870*](https://urldefense.com/v3/__https:/www.science.org/doi/10.1126/sciadv.adv0870__;!!KGKeukY!2gyrR8owd7hrOL3ESwv32OJEiMsxorUn_8nMbkpbo-z_K0fn9zQOLN3_LfNk1clSU-mM6MqQOrZWen0X0p1pMA$)*).*

Response: We thank the reviewer for this technically important comment and for the suggestion to consider RBPSeq as an alternative approach. We fully agree that AlphaFold predictions of elongated fibrous proteins such as phage tail fibers are subject to well-documented limitations in resolving relative domain positioning, and that the curled conformations produced in these predictions do not reliably represent the native extended structure.

We attempted to implement RBPSeq as suggested. Despite sustained and independent efforts by multiple members of our team, including a dedicated bioinformatician in our laboratory, we were unable to generate the required output files from the RBPSeq pipeline when applied to our tail fiber sequences. The tool produced no errors but failed to generate the structural overlap file required for the final merged structure, and we were therefore unable to produce the improved structural comparison the reviewer suggested. We report this transparently so the reviewers and editor can assess the matter fully.

In response to these concerns we have removed the AlphaFold structural predictions from Figure 7 (prior fig panels C,D and E are removed). The evidence for the correlation between tail fiber sequence variation and receptor usage profiles now rests entirely on the phylogenetic tree and multiple sequence alignment, which together clearly demonstrate that the two receptor usage groups defined by RB-TnSeq fitness data segregate by C-terminal sequence identity in the distal tail fiber subunit. We believe this revised presentation is more truthful and does not depend on structural predictions that are difficult to validate for this class of protein.

*Reviewer #2: Table S4. Confirm whether references for cited works in the table are provided somewhere in the text. I could not immediately find them.*

Response: Prior table S4 is now S7, and references were re-checked and cited in the same doc for easier readability.

**—-------------------- Reviewer #3 —---------------------------------**

*Reviewer #3:This manuscript applies the team’s established RB-TnSeq pipeline (applied to E. coli and S. enterica) to identify Klebsiella M5aI genetic determinants for phage infection. They used a capsule-deficient Klebsiella strain and 25 phages. They identified 42 bacterial genes essential for infection, which include surface receptors (such as O antigen), transcriptional regulators, and metabolic and protein-folding pathways. The team validated three O antigen synthesis genes. Based on RB-TnSeq results, the team found that some genes broadly contributed to phage susceptibility, while others only affected susceptibility to a narrow number of phages. They found that bacterial susceptibility factors tended to cluster by phage genus, but there were exceptions.*

Response: We thank Reviewer 3 for the accurate and fair summary of the study and for the positive assessment of the work. We wish to clarify one point in the summary that may reflect ambiguous presentation in the original manuscript. The three validated biosynthesis genes *wabI,* *wabN*, and *wabH* encode enzymes involved in LPS outer core biosynthesis rather than O-antigen synthesis. These are distinct components of the LPS structure, with the outer core representing the conserved inner portion of the lipopolysaccharide molecule assembled proximal to lipid A, while O-antigen refers to the highly variable polysaccharide chain extending distally from the core. The distinction is biologically significant because the outer core is a conserved surface structure across diverse *Klebsiella* strains, whereas O-antigen is highly variable and strain-specific. The validation of outer core biosynthesis genes as phage receptor determinants therefore has broader implications for receptor conservation across *Klebsiella* diversity than O-antigen-dependent receptor usage would. We have ensured that this distinction is stated clearly throughout the revised manuscript to avoid any further confusion between these two components of the LPS structure. We look forward to addressing the reviewer's specific comments below.

*Major:*

*Reviewer #3: 1. Fig 6: If wabH, wabN, or wabI is deleted the sugar moiety added by wabO would also be absent (O antigen would be truncated at the earliest biosynthetic step). Moreover, the waaL and wabJ disruptions would remove all O-antigen, yet these have no fitness cost. Can the authors reconcile these results? Furthermore, the RB-TnSeq results with wabO are not experimentally validated, making these conclusions tenuous.*

Response: We thank the reviewer for this thoughtful comment. We agree that, based on current models of LPS biosynthesis, disruption of *wabH*, *wabN*, or *wabI* would be expected to affect downstream assembly and could result in the absence of the sugar moiety added by WabO. Similarly, disruption of *waaL* or *wabJ* is predicted to abolish O-antigen ligation, leading to a complete loss of surface-exposed O-antigen. However, our RB-TnSeq data reveal a key distinction: insertion in *waaL* and *wabJ* do not confer a measurable fitness advantage under phage challenge. This observation suggests that complete loss of O-antigen is not sufficient to impair phage infection in this system. Instead, these findings are most consistent with a model in which the phages primarily interact with regions of the LPS outside of the O-antigen, most likely within the outer core.

In this context, mutations in *wabH*, *wabN*, or *wabI* would alter outer core structure in a manner that reduces phage adsorption or infection efficiency, while complete removal of O-antigen via *waaL* or *wabJ* disruption would have little to no impact on phage binding. Thus, although upstream disruptions may indirectly affect O-antigen composition, their fitness effects are more parsimoniously explained by structural changes to the outer core rather than by loss of O-antigen per se. We have revised the manuscript to clarify this distinction and to more explicitly separate the contributions of O-antigen and outer core structures.

Despite multiple independent attempts, we were unable to generate a clean deletion mutant of *wabO*. We now state this explicitly in the manuscript and have tempered our interpretation accordingly, presenting the *wabO* result as suggestive rather than conclusive pending future validation.

*Reviewer #3: 2. Line 440-442: The results in Fig 7 do not link the differences in receptors to tail fiber variation. These are correlations that are not experimentally validated. Furthermore, the KLB1 and KLB25 secondary structures predicted by alphaFold look similar. It is possible that alphaFold tertiary structure does not match, but could be similar in a native context, like on a virion.*

Response: We thank the reviewer for this precise and valid observation. The reviewer is correct that Figure 7 as originally presented shows a correlation between tail fiber sequence variation and receptor usage profiles rather than an experimentally validated mechanistic link, and that the secondary structure predictions for KLB1 and KLB25 appear similar, raising the possibility that tertiary structure differences predicted by AlphaFold may not reflect the native conformation on the virion. We have revised the manuscript to address both points directly.

The AlphaFold structural predictions have been removed from Figure 7 in the revised manuscript. The correlation between tail fiber sequence divergence and receptor usage profiles is now presented based solely on the phylogenetic analysis and multiple sequence alignment of the distal tail fiber subunit, which provide clear sequence-level evidence that the two receptor dependency groups segregate by C-terminal domain identity. The Results and Discussion text has been revised to present this clearly as a sequence-based correlation consistent with the RB-TnSeq data rather than as a demonstrated mechanistic link, and we have noted that direct experimental validation of the relationship between tail fiber sequence variation and receptor binding specificity awaits future investigation.

*Reviewer #3: 3. Statistics:
a. Fig 3: Statistical tests applied are not reported.*

Response: Statistical tests are shown in the graph. We also added a sentence in Figure legend and the methods section describing the t-test done using Graphpad.

*Reviewer #3: b. Fig 6D-F: What does the solid black line denote? Are there any statistically significant differences to support conclusions drawn from Fig 6C-F?*

Response: We appreciate the reviewer’s suggestion. EOP heatmaps are typically presented without statistical annotations, as they are intended to provide a comparative and semi-quantitative overview of infection phenotypes rather than support inferential statistical analysis. In our dataset, genes identified as required for infection exhibited a complete loss of plating (below the limit of detection) or a dramatic reduction in EOP.

Specifically, for most validated hits, gene disruption resulted in complete loss of infection. In the few cases where only a partial reduction was observed (KLB4, KLB7, and KLB12), the decrease was less pronounced than complete loss but still corresponded to approximately a 2 log10 reduction in EOP. We have clarified these cases in the revised text to ensure this distinction is clear to readers.

Given that the observed phenotypes consisted of either complete loss or substantial multi-log reductions, we believe that additional statistical annotations on the heatmap would not meaningfully enhance interpretation. However, we have revised the text to more clearly describe the magnitude of the reductions where applicable.

*Minor:
Reviewer #3: 4. Lines 78-79: The intended meaning of this sentence is unclear. The prior sentence details known genetic mechanisms that drive phage susceptibility, but this sentence says genetic mechanisms are unknown. I think the language in this sentence needs to be more precise or expanded to better explain the knowledge gap the authors are trying to describe.*

Response: We clarified the sentence to: “However, these factors do not fully explain the substantial variability in susceptibility observed across strains and phages. In particular, the broader and strain-specific genetic determinants that modulate phage infection beyond well-characterized receptors and defense systems remain incompletely defined”.

*Reviewer #3: 5. Line 142-146: The description of gene fitness in the text does not align with the formula in Fig 1B. In addition, a brief explanation of what the three metrics in Line 146 represent (instead of simply ‘as previously described’) would assist readers in data interpretation.*

Response: Strain fitness was assessed based on the log2 barcode abundance changes from the 4.5 hours after infection initiation over the initial library (“T0”) barcode abundances. The strain fitness for a single gene was then averaged to calculate the gene fitness. Also expanded to better define the metrics, “To be conservative, we restricted our analyses to genes with significantly increased fitness, defined by three criteria: (i) a fitness score ≥ 4, representing the log₂ fold-change in mutant abundance under the tested condition relative to the control; (ii) a t-like statistic ≥ 5, reflecting the consistency of the fitness estimate across independent barcodes and replicates; and (iii) an estimated standard error ≤ 2, indicating high confidence in the calculated fitness value. These thresholds were applied as previously described.”

*Reviewer #3: 6. Line 179: I believe this is intended to refer to Fig 1B*

Response: Thank you for noticing. We changed that line to refer to Figure 1B.

*Reviewer #3: 7. Line 349: Should this state, ‘did not have a gene fitness >4’? Also, I don’t see any proteins with a red border in the figure.*

Response: We have edited this section.

*8. The phage strain order is shuffled in Fig 6C vs Fig 2. It would facilitate reading and data interpretation to keep the phage strains in the same order as much as possible.*

Response: Updated Figure 6C to match the order of Figure 2

*9. Fig 7D-E is not referenced in the text.*

Response: We have revised this figure.
