# Supplementary material for: Cross-family and phage-specific gene requirements for Klebsiella infection revealed by scalable RB-TnSeq genetic screens
Source: PLoS Genet. 2026 Jul 20;22(7):e1012233. doi: 10.1371/journal.pgen.1012233 (PMC13412088; doi:10.1371/journal.pgen.1012233)
Supplement: Attachment — Submitted filename: Response to reviewers_june25-2026.docx [file pgen.1012233.s021.docx]

**Response to reviewers:**

*Reviewer #1: I appreciate the authors detailed and thoughtful responses and for engaging deeply with all of the suggestions, critiques, and comments. I want to acknowledge that they have put in a substantial amount of additional work, and the resulting manuscript is significantly strengthened. This publication will indeed provide a rich and very useful dataset that will be of great value to the field and will lay the groundwork for more in depth mechanistic studies.
I have just one quibble with a response to one of the points I made regarding that fact that this screen is unlikely to identify defense systems (which was born out by the data). The authors make two reasonable points: 1) if a defense system targets a phage, then it will be evident in a Tn-seq experiment; 2) most defense systems have been studied under non-native expression conditions. One possibility thus is that defense systems are generally a smaller factor in phage fitness if most are less potent than many papers suggest, which is what the authors are proposing. I don’t necessarily disagree with this, however, the design of this screen is not equipped to answer this question for the following reason: By isolating phages on a particular host and using only these phages in the screen, they have selected for phages that are not strongly targeted by the defense systems found in this host. Defense systems vary dramatically among even closely related strains of a species. The screen design is thus is biased against finding defense systems as the phages used are by definition not (fully) blocked by any defense systems in this strain. If they wanted to find defense systems with this method (which is not necessarily something I would recommend), they would need to screen with phages that have compatible receptor binding proteins but that are unable to plaque well on this host. As a result, this dataset cannot shed light on the larger role defense systems play in defining host range. I do think this is a caveat they should acknowledge in either the introduction or conclusion given their statement in the introduction to motivate their study, “In particular, the broader and strain-specific genetic determinants that modulate phage infection beyond well-characterized receptors and defense systems remain incompletely defined”.*

We thank the reviewer for this thoughtful comment and agree regarding the important caveat pointed out.

We have added the following sentence to “Non-receptor host factors uncovered in RB-TnSeq screens’ section at

line number # 428 "Finally, we note that because the phages used in this screen were isolated on Klebsiella sp. M5al, they have by definition overcome or evaded the defense systems present in this strain; disruption of those defense genes would confer no fitness advantage in the RB-TnSeq library, and this screen design is therefore not suited to evaluating the contribution of defense systems to host range in this strain."

*Reviewer #2: The authors have adequately addressed my comments. They clarified and nuanced their work, and performed additional knockout/complementation work. The re-focus to the high-throughput screening is a safe route. I found no further issues in the substantially revised work apart some minor elements:
Line 932 and 934: dsbA should be in italic*

Done

*Ref 91 is published in 2022 instead of 2021*

Done
*Table S15 with primers seems to be missing in the submission*

Done
*Further I would have appreciated if the authors would have added line numbers in the response letter to facilitate identification/location of the specific changes.*

Apologies for this. We have fixed it this time.

*Reviewer #3: Thank you for providing line numbers for the edits made.
The authors have been reasonably responsive to my prior concerns.

Except for the following comment:

Reviewer #3: 2. Line 440-442: The results in Fig 7 do not link the differences in receptors to tail fiber variation. These are correlations that are not experimentally validated. Furthermore, the KLB1 and KLB25 secondary structures predicted by alphaFold look similar. It is possible that alphaFold tertiary structure does not match, but could be similar in a native context, like on a virion. Response: AlphaFold panels removed. The Results and Discussion now present the tail fiber — receptor relationship explicitly as a sequence-based correlation.
The revised text states: Line 361"These data indicate that intra-genus divergence in receptor usage is driven by sequence variation in the distal tail fiber, and that subtle genetic variation among closely related phages can produce functionally distinct host interactions" --> The conclusions are still drawn based on sequence similarity and not experimentally validated. The author ought to acknowledge this limitation in their conclusion. This could be accomplished with minor text edits: "These data suggest that...receptor usage is likely driven by...phages likely produces functionally...."*

We thank the reviewer for this comment. We agree that the conclusion drawn at line 361 overstates the evidence, as the relationship between tail fiber sequence variation and receptor usage differences is based on sequence correlation rather than experimental validation. The sentence has been revised as follows: line 361

"These data suggest that intra-genus divergence in receptor usage is likely driven by sequence variation in the distal tail fiber, and that subtle genetic variation among closely related phages likely produces functionally distinct host interactions."
