# Supplementary material for: Cross-family and phage-specific gene requirements for Klebsiella infection revealed by scalable RB-TnSeq genetic screens
Source: PLoS Genet. 2026 Jul 20;22(7):e1012233. doi: 10.1371/journal.pgen.1012233 (PMC13412088; doi:10.1371/journal.pgen.1012233)
Supplement: Peer review history

# Author comment: Author response to Decision Letter 0

**DOI:** 10.1371/journal.pgen.1012233.r001

**Transfer Alert**

This paper was transferred from another journal. As a result, its full editorial history (including decision letters, peer reviews and author responses) may not be present.

10 Nov 2025

# Peer review: Decision Letter 0

**DOI:** 10.1371/journal.pgen.1012233.r002

**Authors:** Sean Crosson, Shumin Tan

23 Dec 2025

PGENETICS-D-25-01196

Cross-family and phage-specific gene requirements for Klebsiella infection revealed by scalable RB-TnSeq genetic screens

PLOS Genetics

Dear Dr. Sullivan,

Thank you for submitting your manuscript to PLOS Genetics. After careful consideration, we feel that it has merit but does not fully meet PLOS Genetics's publication criteria as it currently stands. Therefore, we invite you to submit a revised version of the manuscript that addresses the points raised during the review process.

Please submit your revised manuscript by Feb 21 2026 11:59PM. If you will need more time than this to complete your revisions, please reply to this message or contact the journal office at plosgenetics@plos.org. When you're ready to submit your revision, log on to https://www.editorialmanager.com/pgenetics/ and select the 'Submissions Needing Revision' folder to locate your manuscript file.

Please include the following items when submitting your revised manuscript:

* A letter that responds to each point raised by the editor and reviewer(s). You should upload this letter as a separate file labeled 'Response to Reviewers'. This file does not need to include responses to any formatting updates and technical items listed in the 'Journal Requirements' section below.

* A marked-up copy of your manuscript that highlights changes made to the original version. You should upload this as a separate file labeled 'Revised Manuscript with Track Changes'.

* An unmarked version of your revised paper without tracked changes. You should upload this as a separate file labeled 'Manuscript'.

If you would like to make changes to your financial disclosure, competing interests statement, or data availability statement, please make these updates within the submission form at the time of resubmission. Guidelines for resubmitting your figure files are available below the reviewer comments at the end of this letter.

We look forward to receiving your revised manuscript.

Kind regards,

Shumin Tan, Ph.D.

Academic Editor

PLOS Genetics

Sean Crosson

Section Editor

PLOS Genetics

Aimée Dudley

Editor-in-Chief

PLOS Genetics

Anne Goriely

Editor-in-Chief

PLOS Genetics

**Additional Editor Comments:**

While the application of RB-TnSeq to Klebsiella and the work that went into this study was appreciated by the reviewers, several limitations were raised. In particular, a resubmission should include additional experiments following up on at least a few hits that were not aspects already well understood to be involved in phage infection of Klebsiella, to validate new biological insight.

**Journal Requirements:**

If the reviewer comments include a recommendation to cite specific previously published works, please review and evaluate these publications to determine whether they are relevant and should be cited. There is no requirement to cite these works unless the editor has indicated otherwise.

1) Some material included in your submission may be copyrighted. According to PLOSu2019s copyright policy, authors who use figures or other material (e.g., graphics, clipart, maps) from another author or copyright holder must demonstrate or obtain permission to publish this material under the Creative Commons Attribution 4.0 International (CC BY 4.0) License used by PLOS journals. Please closely review the details of PLOSu2019s copyright requirements here: PLOS Licenses and Copyright. If you need to request permissions from a copyright holder, you may use PLOS's Copyright Content Permission form.

Please respond directly to this email and provide any known details concerning your material's license terms and permissions required for reuse, even if you have not yet obtained copyright permissions or are unsure of your material's copyright compatibility. Once you have responded and addressed all other outstanding technical requirements, you may resubmit your manuscript within Editorial Manager.

Potential Copyright Issues:

i) Figure 1B. Please confirm whether you drew the images / clip-art within the figure panels by hand. If you did not draw the images, please provide (a) a link to the source of the images or icons and their license / terms of use; or (b) written permission from the copyright holder to publish the images or icons under our CC BY 4.0 license. Alternatively, you may replace the images with open source alternatives. See these open source resources you may use to replace images / clip-art:

- https://commons.wikimedia.org

- https://openclipart.org/.

2) In the online submission form, you indicated that ll phage stocks are available from the Félix d'Hérelle Reference Center for bacterial viruses of the Université Laval or by contacting the corresponding author Matthew Sullivan at OSU.. All PLOS journals now require all data underlying the findings described in their manuscript to be freely available to other researchers, either

1. In a public repository

2. Within the manuscript itself

3. Uploaded as supplementary information.

This policy applies to all data except where public deposition would breach compliance with the protocol approved by your research ethics board. If your data cannot be made publicly available for ethical or legal reasons (e.g., public availability would compromise patient privacy), please explain your reasons by return email and your exemption request will be escalated to the editor for approval. Your exemption request will be handled independently and will not hold up the peer review process, but will need to be resolved should your manuscript be accepted for publication. One of the Editorial team will then be in touch if there are any issues.

3) Kindly revise your competing statement in the online submission form to align with the journal's style guidelines: 'The authors declare that there are no competing interests.'

**Reviewers' comments:**

Reviewer's Responses to Questions

**Comments to the Authors:**

**Please note here if the review is uploaded as an attachment.**

Reviewer #1: In “Cross-family and phage-specific gene requirements for Klebsiella infection revealed by scalable RB-TnSeq genetic screens”, Gittrich et al. perform a series of barcoded Tn-seq screens to identify fitness determinants for a panel of 25 dsDNA Klebsiella phages that infect the acapsular environmental Klebsiella species M15aI. The stated goal of the study was to identify genes required for infection of each phage and from there to discern broader patterns comparing across the diverse phages. This type of screen is successful at identifying phage receptors or genes that encode regulators of phage receptors, as has been seen in previous similar efforts. Most other bacterial genes required for phage replication are likely essential, e.g. any of the central dogma processes that phages hijack and commandeer to replicate themselves. A screen like this will also not identify any genes involved in bacterial antiphage defenses – which also contribute to host range – as the phages used here are by definition are not susceptible to any defenses present in this particular strain. Thus, as expected, most of the 42 hits identified here are in genes that are already associated with phage infection, as these are directly or indirectly involved in producing or regulating production of phage receptors. The authors do not follow up at all on any of the unexpected genes that they hit. From a technical standpoint, the Tn-seq has been carefully carried out with sufficient replicates, and clearly represents a laborious effort. However, despite the scale of the screen, it does not appear we have learned much new biology. One lens through which this work could be viewed is as a high throughput method for mapping phage receptors; however, the authors do not take this angle. I also do not think that based on this study we can conclude very much about infection strategies or requirements (outside of receptors), as many of the genes required for infection – as I noted above – are likely essential. Yet as I elaborate on below, of the potentially unexpected genes identified, none were followed up on in much depth.

There are a few interesting hits that are not obviously receptors: NlpD, TolR. The authors discuss possible well thought through and referenced hypotheses for each at length, but not only do they not test any of these, they do not even validate these hits via knock out or complementation. Similarly, regarding the regulators they hit, they speculate that e.g. SdiA might alter phage adsorption via increases in biofilm formation when it is deleted; however, this is never actually tested. One hit that was not an obvious receptor is DsbA, but notably, DsbA was identified in an earlier, similar Tn-seq study performed in Acinetobacter where the authors delved into how it affects capsule synthesis; here, we have no further information about what might be happening. It seems potentially interesting that DsbA was hit in a study with a capsulated bacterium as well as here; do they signal through a similar regulator that alters the cell surface here as well? Finally, they identify 3 genes never before associated with phage infection – a stated objective of their study – but do not follow up on these at all. None of hits, except for DsbA and WabI, were validated in single gene deletion assays or through complementation. They perform some analyses looking for patterns among and between phages, and find that consistent with many previous studies, phage receptor usage mediated by phage tail fiber variation is responsible for most of the variation observed. Thus despite their contention that their work with acapsular strains can identify a broader set of host range determinants, which may or may not be the case, they did not really leverage this stated advantage to learn anything more about these determinants.

Minor point: the figures were extremely pixelated to the point that it was hard to interpret the data in some cases (e.g. the PCA analysis I could barely tell apart the different shapes of the same color).

Reviewer #2: Using a genome-wide RB-TnSeq approach in Klebsiella sp. M5al, this study identifies 42 bacterial genes required for phage infection across diverse phage families. The work demonstrates that bacterial gene requirements cluster significantly by phage taxonomy, while revealing that even closely related phages can exhibit distinct infection strategies due to tail fiber variation and differential reliance on intracellular host factors. The manuscript is original, well written, and supported by extensive raw and processed data.

Specific comments

• Line 76: The term phage tail fibers is too restrictive, as not all receptor-binding proteins adopt a fibrous morphology (e.g., tail spikes). Phage receptor-binding proteins would be a more accurate general term.

• Line 96: Please elaborate on the RB-TnSeq principle for readers unfamiliar with the method. Note that if a phage depends on a bacterial protein essential for host survival, this dependency cannot be detected with RB-TnSeq. This limitation seems currently not acknowledged. Additionally, the dataset reflects infections in rich medium; nutrient composition likely influences host gene dependencies. In nutrient-poor conditions, dependencies may increase, but more bacterial genes will become essential and can’t be discovered with this approach. Was a phage-free control included over the same time interval? Some enriched mutants, particularly those difficult to interpret, might reflect general growth advantages under the experimental conditions (LB, shaking, 30°C). These methodological limitations should be explicitly discussed.

• Line 135: Justify the selection of the 24 + 1 phages. The choice appears pragmatic based on availability (which is understandable) rather than diversity-driven (e.g., absence of Casjensviridae or Peduoviridae; see Fig. 1).

• Discussion on Autographiviridae: These phages are often described as relatively host-independent due to their RNA polymerase, which explains their frequent use in engineering projects for host swapping. This trend is partially reflected in the dataset, with the three Autographiviridae showing reduced dependence on intracellular proteins compared to Drexlerviridae and some Straboviridae. However, RS11475 is an exception for Autographiviridae and is not discussed.

• Validation of RB-TnSeq hits: Not all targets identified and discussed are validated by knockouts (ideally complemented to exclude polar effects). The manuscript uses the term “essential” for all 42 genes (e.g. in the abstract), yet several hits confer only partial resistance and lack orthogonal validation. Essential genes imply strict necessity for infection, requiring absolute functional requirement. Figures 2 and 3 suggest context- and phage-specific fitness signals, with dsbA disruption yielding modest effects, indicating graded rather than absolute requirements. Without systematic single-gene knockouts and complementation, essentiality cannot be conclusively established.

• Line 410–411: The statement does not hold for KLB12 and wbiN/wbiH. Please clarify the suggestion for additional receptor usage and how this claim is derived. An additional receptor typically refers to a secondary receptor engaged after initial binding to the primary receptor. Do the authors mean two possible primary receptors instead?

• Line 437: Clarify that “the protein itself” refers to the distal tip of the long tail fiber, not one of the three other conserved long tail fiber proteins. For reproducibility, include accession numbers for these proteins in lines 716–721. The discussion could be strengthened: the “red” KLB15 tip domain is overall more similar to the “blue” tip, suggesting horizontal transfer of the C-terminal domain, which ultimately interacts with LPS, correlating with the binding profiles.

• Figure 7: AlphaFold predictions suffer from its inability to resolve relative domain positioning, resulting in curling rather than straight fibrous structures in this case. Quality metrics (pLDDT, PAE) for these predictions are missing, and the superimposition of both tail fibers is problematic. Consider alternative approaches such as RBPSeq (https://www.science.org/doi/10.1126/sciadv.adv0870).

• Table S4: Confirm whether references for cited works in the table are provided somewhere in the text. I could not immediately find them.

Reviewer #3: This manuscript applies the team’s established RB-TnSeq pipeline (applied to E. coli and S. enterica) to identify Klebsiella M5aI genetic determinants for phage infection. They used a capsule-deficient Klebsiella strain and 25 phages. They identified 42 bacterial genes essential for infection, which include surface receptors (such as O antigen), transcriptional regulators, and metabolic and protein-folding pathways. The team validated three O antigen synthesis genes. Based on RB-TnSeq results, the team found that some genes broadly contributed to phage susceptibility, while others only affected susceptibility to a narrow number of phages. They found that bacterial susceptibility factors tended to cluster by phage genus, but there were exceptions.

Major:

1. Fig 6: If wabH, wabN, or wabI is deleted the sugar moiety added by wabO would also be absent (O antigen would be truncated at the earliest biosynthetic step). Moreover, the waaL and wabJ disruptions would remove all O-antigen, yet these have no fitness cost. Can the authors reconcile these results? Furthermore, the RB-TnSeq results with wabO are not experimentally validated, making these conclusions tenuous.

2. Line 440-442: The results in Fig 7 do not link the differences in receptors to tail fiber variation. These are correlations that are not experimentally validated. Furthermore, the KLB1 and KLB25 secondary structures predicted by alphaFold look similar. It is possible that alphaFold tertiary structure does not match, but could be similar in a native context, like on a virion.

3. Statistics:

a. Fig 3: Statistical tests applied are not reported.

b. Fig 6D-F: What does the solid black line denote? Are there any statistically significant differences to support conclusions drawn from Fig 6C-F?

Minor:

4. Lines 78-79: The intended meaning of this sentence is unclear. The prior sentence details known genetic mechanisms that drive phage susceptibility, but this sentence says genetic mechanisms are unknown. I think the language in this sentence needs to be more precise or expanded to better explain the knowledge gap the authors are trying to describe.

5. Line 142-146: The description of gene fitness in the text does not align with the formula in Fig 1B. In addition, a brief explanation of what the three metrics in Line 146 represent (instead of simply ‘as previously described’) would assist readers in data interpretation.

6. Line 179: I believe this is intended to refer to Fig 1B

7. Line 349: Should this state, ‘did not have a gene fitness >4’? Also, I don’t see any proteins with a red border in the figure.

8. The phage strain order is shuffled in Fig 6C vs Fig 2. It would facilitate reading and data interpretation to keep the phage strains in the same order as much as possible.

9. Fig 7D-E is not referenced in the text.

**********

**Have all data underlying the figures and results presented in the manuscript been provided?**

Large-scale datasets should be made available via a public repository as described in the *PLOS Genetics*  data availability policy, and numerical data that underlies graphs or summary statistics should be provided in spreadsheet form as supporting information.

Reviewer #1: Yes

Reviewer #2: Yes

Reviewer #3: Yes

**********

PLOS authors have the option to publish the peer review history of their article (what does this mean?). If published, this will include your full peer review and any attached files.

If you choose “no”, your identity will remain anonymous but your review may still be made public.

**Do you want your identity to be public for this peer review?**  For information about this choice, including consent withdrawal, please see our Privacy Policy.

Reviewer #1: No

Reviewer #2: **Yes:**  Yves Briers

Reviewer #3: No

**Figure resubmission:**

While revising your submission, we strongly recommend that you use PLOS’s NAAS tool (https://ngplosjournals.pagemajik.ai/artanalysis) to test your figure files. NAAS can convert your figure files to the TIFF file type and meet basic requirements (such as print size, resolution), or provide you with a report on issues that do not meet our requirements and that NAAS cannot fix.

After uploading your figures to PLOS’s NAAS tool - https://ngplosjournals.pagemajik.ai/artanalysis, NAAS will process the files provided and display the results in the "Uploaded Files" section of the page as the processing is complete. If the uploaded figures meet our requirements (or NAAS is able to fix the files to meet our requirements), the figure will be marked as "fixed" above. If NAAS is unable to fix the files, a red "failed" label will appear above. When NAAS has confirmed that the figure files meet our requirements, please download the file via the download option, and include these NAAS processed figure files when submitting your revised manuscript.

**Reproducibility:**

To enhance the reproducibility of your results, we recommend that authors of applicable studies deposit laboratory protocols in protocols.io, where a protocol can be assigned its own identifier (DOI) such that it can be cited independently in the future. Additionally, PLOS ONE offers an option to publish peer-reviewed clinical study protocols. Read more information on sharing protocols at https://plos.org/protocols?utm_medium=editorial-email&utm_source=authorletters&utm_campaign=protocols

# Author comment: Author response to Decision Letter 1

**DOI:** 10.1371/journal.pgen.1012233.r003

8 May 2026

Attachment Submitted filename: Response to reviewers.docx

# Peer review: Decision Letter 1

**DOI:** 10.1371/journal.pgen.1012233.r004

**Authors:** Sean Crosson, Shumin Tan

17 Jun 2026

PGENETICS-D-25-01196R1

Cross-family and phage-specific gene requirements for Klebsiella infection revealed by scalable RB-TnSeq genetic screens

PLOS Genetics

Dear Dr. Mutalik,

Thank you for submitting your manuscript to PLOS Genetics. After careful consideration, we feel that it has merit but does not fully meet PLOS Genetics's publication criteria as it currently stands. Therefore, we invite you to submit a revised version of the manuscript that addresses the points raised during the review process.

Please submit your revised manuscript within by Jul 17 2026 11:59PM. If you will need more time than this to complete your revisions, please reply to this message or contact the journal office at plosgenetics@plos.org. When you're ready to submit your revision, log on to https://www.editorialmanager.com/pgenetics/ and select the 'Submissions Needing Revision' folder to locate your manuscript file.

Please include the following items when submitting your revised manuscript:

* A letter that responds to each point raised by the editor and reviewer(s). You should upload this letter as a separate file labeled 'Response to Reviewers'. This file does not need to include responses to formatting updates and technical items listed in the 'Journal Requirements' section below.

* A marked-up copy of your manuscript that highlights changes made to the original version. You should upload this as a separate file labeled 'Revised Manuscript with Track Changes'.

* An unmarked version of your revised paper without tracked changes. You should upload this as a separate file labeled 'Manuscript'.

If you would like to make changes to your financial disclosure, competing interests statement, or data availability statement, please make these updates within the submission form at the time of resubmission. Guidelines for resubmitting your figure files are available below the reviewer comments at the end of this letter.

As the corresponding author, your ORCID iD is verified in the submission system and will appear in the published article. PLOS supports the use of ORCID, and we encourage all coauthors to register for an ORCID iD and use it as well. Please encourage your coauthors to verify their ORCID iD within the submission system before final acceptance, as unverified ORCID iDs will not appear in the published article. Only the individual author can complete the verification step; PLOS staff cannot verify ORCID iDs on behalf of authors.

We look forward to receiving your revised manuscript.

Kind regards,

Shumin Tan, Ph.D.

Academic Editor

PLOS Genetics

Sean Crosson

Section Editor

PLOS Genetics

Aimée Dudley

Editor-in-Chief

PLOS Genetics

Anne Goriely

Editor-in-Chief

PLOS Genetics

**Additional Editor Comments (if provided):**

All reviewers appreciated the thoughtful revision, and there are just a few remaining comments that should be addressed.

**Journal Requirements:**

If the reviewer comments include a recommendation to cite specific previously published works, please review and evaluate these publications to determine whether they are relevant and should be cited. There is no requirement to cite these works unless the editor has indicated otherwise.

1) We have noticed that you have a list of Supporting Information legends For Table S15 in your manuscript. However, there are no corresponding files uploaded to the submission. Please upload them as separate files with the item type 'Supporting Information'.

2) In the online submission form, you indicated that All 25 phage genomes isolated in this study are available from the NCBI database (accession numbers PP197198, PP197344, PP202993, PP444685, PP44686 PP44687, PP4688, PP505786, PP505787, PP526028, PP526029, PP554336, PP554337, PP554338, PP56864, PP556865, PP582754, PP582755, PP582756, PP582757, PP582758, PP597382, PP597383, PP597384, PX412413) All phage stocks are available from the Félix d'Hérelle Reference Center for bacterial viruses of the Université Laval or by contacting the corresponding author Matthew Sullivan at OSU.. All PLOS journals now require all data underlying the findings described in their manuscript to be freely available to other researchers, either

1. In a public repository

2. Within the manuscript itself

3. Uploaded as supplementary information.

This policy applies to all data except where public deposition would breach compliance with the protocol approved by your research ethics board. If your data cannot be made publicly available for ethical or legal reasons (e.g., public availability would compromise patient privacy), please explain your reasons by return email and your exemption request will be escalated to the editor for approval. Your exemption request will be handled independently and will not hold up the peer review process, but will need to be resolved should your manuscript be accepted for publication. One of the Editorial team will then be in touch if there are any issues.

**Reviewers' comments:**

Reviewer's Responses to Questions

**Comments to the Authors:**

**Please note here if the review is uploaded as an attachment.**

Reviewer #1: I appreciate the authors detailed and thoughtful responses and for engaging deeply with all of the suggestions, critiques, and comments. I want to acknowledge that they have put in a substantial amount of additional work, and the resulting manuscript is significantly strengthened. This publication will indeed provide a rich and very useful dataset that will be of great value to the field and will lay the groundwork for more in depth mechanistic studies.

I have just one quibble with a response to one of the points I made regarding that fact that this screen is unlikely to identify defense systems (which was born out by the data). The authors make two reasonable points: 1) if a defense system targets a phage, then it will be evident in a Tn-seq experiment; 2) most defense systems have been studied under non-native expression conditions. One possibility thus is that defense systems are generally a smaller factor in phage fitness if most are less potent than many papers suggest, which is what the authors are proposing. I don’t necessarily disagree with this, however, the design of this screen is not equipped to answer this question for the following reason:

By isolating phages on a particular host and using only these phages in the screen, they have selected for phages that are not strongly targeted by the defense systems found in this host. Defense systems vary dramatically among even closely related strains of a species. The screen design is thus is biased against finding defense systems as the phages used are by definition not (fully) blocked by any defense systems in this strain. If they wanted to find defense systems with this method (which is not necessarily something I would recommend), they would need to screen with phages that have compatible receptor binding proteins but that are unable to plaque well on this host. As a result, this dataset cannot shed light on the larger role defense systems play in defining host range. I do think this is a caveat they should acknowledge in either the introduction or conclusion given their statement in the introduction to motivate their study, “In particular, the broader and strain-specific genetic determinants that modulate phage infection beyond well-characterized receptors and defense systems remain incompletely defined”.

Reviewer #2: The authors have adequately addressed my comments. They clarified and nuanced their work, and performed additional knockout/complementation work. The re-focus to the high-throughput screening is a safe route. I found no further issues in the substantially revised work apart some minor elements:

Line 932 and 934: dsbA should be in italic

Ref 91 is published in 2022 instead of 2021

Table S15 with primers seems to be missing in the submission

Further I would have appreciated if the authors would have added line numbers in the response letter to facilitate identification/location of the specific changes.

Reviewer #3: Thank you for providing line numbers for the edits made.

The authors have been reasonably responsive to my prior concerns.

Except for the following comment:

Reviewer #3: 2. Line 440-442: The results in Fig 7 do not link the differences in receptors to tail fiber variation. These are correlations that are not experimentally validated. Furthermore, the KLB1 and KLB25 secondary structures predicted by alphaFold look similar. It is possible that alphaFold tertiary structure does not match, but could be similar in a native context, like on a virion.

Response: AlphaFold panels removed. The Results and Discussion now present the tail fiber — receptor relationship explicitly as a sequence-based correlation.

The revised text states: Line 361

"These data indicate that intra-genus divergence in receptor usage is driven by sequence variation in the distal tail fiber, and that subtle genetic variation among closely related phages can produce functionally distinct host interactions"

--> The conclusions are still drawn based on sequence similarity and not experimentally validated. The author ought to acknowledge this limitation in their conclusion. This could be accomplished with minor text edits:

"These data suggest that...receptor usage is likely driven by...phages likely produces functionally...."

**********

**Have all data underlying the figures and results presented in the manuscript been provided?**

Large-scale datasets should be made available via a public repository as described in the *PLOS Genetics*  data availability policy, and numerical data that underlies graphs or summary statistics should be provided in spreadsheet form as supporting information.

Reviewer #1: Yes

Reviewer #2: Yes

Reviewer #3: **No:**  There is no data availability statement provided and it is unclear whether it is provided in one of the 17 supplemental files or not.

**********

PLOS authors have the option to publish the peer review history of their article (what does this mean?). If published, this will include your full peer review and any attached files.

If you choose “no”, your identity will remain anonymous but your review may still be made public.

**Do you want your identity to be public for this peer review?**  For information about this choice, including consent withdrawal, please see our Privacy Policy.

Reviewer #1: No

Reviewer #2: No

Reviewer #3: No

[NOTE: If reviewer comments were submitted as an attachment file, they will be attached to this email and accessible via the submission site. Please log into your account, locate the manuscript record, and check for the action link "View Attachments". If this link does not appear, there are no attachment files.]

**Figure resubmission:**

-->While revising your submission, we strongly recommend that you use PLOS’s NAAS tool (https://ngplosjournals.pagemajik.ai/artanalysis) to test your figure files. NAAS can convert your figure files to the TIFF file type and meet basic requirements (such as print size, resolution), or provide you with a report on issues that do not meet our requirements and that NAAS cannot fix.

After uploading your figures to PLOS’s NAAS tool - https://ngplosjournals.pagemajik.ai/artanalysis, NAAS will process the files provided and display the results in the "Uploaded Files" section of the page as the processing is complete. If the uploaded figures meet our requirements (or NAAS is able to fix the files to meet our requirements), the figure will be marked as "fixed" above. If NAAS is unable to fix the files, a red "failed" label will appear above. When NAAS has confirmed that the figure files meet our requirements, please download the file via the download option, and include these NAAS processed figure files when submitting your revised manuscript.

**Reproducibility:**

To enhance the reproducibility of your results, we recommend that authors deposit laboratory protocols in protocols.io, where a protocol can be assigned its own identifier (DOI) such that it can be cited independently in the future. Additionally, PLOS ONE offers an option to publish peer-reviewed clinical study protocols. Read more information on sharing protocols at https://plos.org/protocols?utm_medium=editorial-email&utm_source=authorletters&utm_campaign=protocols

# Author comment: Author response to Decision Letter 2

**DOI:** 10.1371/journal.pgen.1012233.r005

26 Jun 2026

Attachment Submitted filename: Response to reviewers_june25-2026.docx

# Editor report: Decision Letter 2

**DOI:** 10.1371/journal.pgen.1012233.r006

**Authors:** Sean Crosson, Shumin Tan

1 Jul 2026

Dear Dr Mutalik,

We are pleased to inform you that your manuscript entitled "Cross-family and phage-specific gene requirements for Klebsiella infection revealed by scalable RB-TnSeq genetic screens" has been editorially accepted for publication in PLOS Genetics. Congratulations!

Before your submission can be formally accepted and sent to production you will need to complete our formatting changes, which you will receive in a follow up email. Please be aware that it may take several days for you to receive this email; during this time no action is required by you. Please note: the accept date on your published article will reflect the date of this provisional acceptance, but your manuscript will not be scheduled for publication until the required changes have been made.

Once your paper is formally accepted, an uncorrected proof of your manuscript will be published online ahead of the final version, unless you’ve already opted out via the online submission form. If, for any reason, you do not want an earlier version of your manuscript published online or are unsure if you have already indicated as such, please let the journal staff know immediately at plosgenetics@plos.org.

In the meantime, please log into Editorial Manager at https://www.editorialmanager.com/pgenetics/, click the "Update My Information" link at the top of the page, and update your user information to ensure an efficient production and billing process. Note that PLOS requires an ORCID iD for all corresponding authors. Therefore, please ensure that you have an ORCID iD and that it is validated in Editorial Manager. To do this, go to ‘Update my Information’ (in the upper left-hand corner of the main menu), and click on the Fetch/Validate link next to the ORCID field.  This will take you to the ORCID site and allow you to create a new iD or authenticate a pre-existing iD in Editorial Manager.

If you have a press-related query, or would like to know about making your underlying data available (as you will be aware, this is required for publication), please see the end of this email. If your institution or institutions have a press office, please notify them about your upcoming article at this point, to enable them to help maximise its impact. Inform journal staff as soon as possible if you are preparing a press release for your article and need a publication date.

Thank you again for supporting open-access publishing; we are looking forward to publishing your work in PLOS Genetics!

Yours sincerely,

Shumin Tan, Ph.D.

Academic Editor

PLOS Genetics

Sean Crosson

Section Editor

PLOS Genetics

Aimée Dudley

Editor-in-Chief

PLOS Genetics

Anne Goriely

Editor-in-Chief

PLOS Genetics

www.plosgenetics.org

BlueSky: @plos.bsky.social

----------------------------------------------------

Comments from the reviewers (if applicable):

----------------------------------------------------

**Data Deposition**

If you have submitted a Research Article or Front Matter that has associated data that are not suitable for deposition in a subject-specific public repository (such as GenBank or ArrayExpress), one way to make that data available is to deposit it in the Dryad Digital Repository. As you may recall, we ask all authors to agree to make data available; this is one way to achieve that. A full list of recommended repositories can be found on our website.

The following link will take you to the Dryad record for your article, so you won't have to re‐enter its bibliographic information, and can upload your files directly:

http://datadryad.org/submit?journalID=pgenetics&manu=PGENETICS-D-25-01196R2

More information about depositing data in Dryad is available at http://www.datadryad.org/depositing. If you experience any difficulties in submitting your data, please contact help@datadryad.org for support.

Additionally, please be aware that our data availability policy requires that all numerical data underlying display items are included with the submission, and you will need to provide this before we can formally accept your manuscript, if not already present.

----------------------------------------------------

**Press Queries**

If you or your institution will be preparing press materials for this manuscript, or if you need to know your paper's publication date for media purposes, please inform the journal staff as soon as possible so that your submission can be scheduled accordingly. Your manuscript will remain under a strict press embargo until the publication date and time. This means an early version of your manuscript will not be published ahead of your final version. PLOS Genetics may also choose to issue a press release for your article. If there's anything the journal should know or you'd like more information, please get in touch via plosgenetics@plos.org.

# Editor report: Acceptance letter

**DOI:** 10.1371/journal.pgen.1012233.r007

**Authors:** Sean Crosson, Shumin Tan

PGENETICS-D-25-01196R2

Cross-family and phage-specific gene requirements for Klebsiella infection revealed by scalable RB-TnSeq genetic screens

Dear Dr Mutalik,

We are pleased to inform you that your manuscript entitled "Cross-family and phage-specific gene requirements for Klebsiella infection revealed by scalable RB-TnSeq genetic screens" has been formally accepted for publication in PLOS Genetics! Your manuscript is now with our production department and you will be notified of the publication date in due course.

The corresponding author will soon be receiving a typeset proof for review, to ensure errors have not been introduced during production. Please review the PDF proof of your manuscript carefully, as this is the last chance to correct any errors. Please note that major changes, or those which affect the scientific understanding of the work, will likely cause delays to the publication date of your manuscript.

Soon after your final files are uploaded, unless you have opted out or your manuscript is a front-matter piece, the early version of your manuscript will be published online. The date of the early version will be your article's publication date. The final article will be published to the same URL, and all versions of the paper will be accessible to readers.

For Research Articles, you will receive an invoice from PLOS for your publication fee after your manuscript has reached the completed accept phase. If you receive an email requesting payment before acceptance or for any other service, this may be a phishing scheme. Learn how to identify phishing emails and protect your accounts at https://explore.plos.org/phishing.

Thank you again for supporting PLOS Genetics and open-access publishing. We are looking forward to publishing your work!

With kind regards,

Kannan R K Kuppusamy, B.TECH BIOTECHNOLOGY

PLOS Genetics

On behalf of:

The PLOS Genetics Team

Carlyle House, Carlyle Road, Cambridge CB4 3DN | United Kingdom

plosgenetics@plos.org | +44 (0) 1223-442823

plosgenetics.org | Twitter: @PLOSGenetics
